# Pan-cortical electrophysiologic changes underlying attention

**DOI:** 10.1038/s41598-024-52717-w

**Published:** 2024-02-01

**Authors:** Ronald P. Lesser, W. R. S. Webber, Diana L. Miglioretti

**Affiliations:** 1 Department of Neurology, Johns Hopkins University School of Medicine, Baltimore, MD 21287 USA; 2 Department of Neurological Surgery, Johns Hopkins University School of Medicine, Baltimore, MD 21287 USA; 3 Department of Public Health Sciences, Davis, School of Medicine, University of California, Davis, CA 95616 USA; 4 Kaiser Permanente Washington Health Research Institute, Seattle, WA 98101 USA

**Keywords:** Cognitive neuroscience, Computational neuroscience, Neurology

## Abstract

We previously reported that pan-cortical effects occur when cognitive tasks end afterdischarges. For this report, we analyzed wavelet cross-coherence changes during cognitive tasks used to terminate afterdischarges studying multiple time segments and multiple groups of inter-electrode-con distances. We studied 12 patients with intractable epilepsy, with 970 implanted electrode contacts, and 39,871 electrode contact combinations. When cognitive tasks ended afterdischarges, coherence varied similarly across the cortex throughout the tasks, but there were gradations with time, distance, and frequency: (1) They tended to progressively decrease relative to baseline with time and then to increase toward baseline when afterdischarges ended. (2) During most time segments, decreases from baseline were largest for the closest inter-contact distances, moderate for intermediate inter-contact distances, and smallest for the greatest inter-contact distances. With respect to our patients’ intractable epilepsy, the changes found suggest that future therapies might treat regions beyond those closest to regions of seizure onset and treat later in a seizure’s evolution. Similar considerations might apply to other disorders. Our findings also suggest that cognitive tasks can result in pan-cortical coherence changes that participate in underlying attention, perhaps complementing the better-known regional mechanisms.

## Introduction

Seven decades ago, Penfield and Jasper noted that epileptiform activity could be terminated by “attention, that is, when the patient is asked a question, or when he is solving an arithmetical problem”^1^. We have reported that brief pulse stimulation (BPS) can end afterdischarges (AD), epileptiform discharges occurring during cortical stimulation testing, but that this is not always successful^2^. In accord with the observations by Penfield and Jasper^1^, we found that arithmetic and spelling-related cognitive tasks (AST) could terminate afterdischarges even when BPS was not successful. There were several underlying correlations. First^3^, local brain states during these tasks could correlate with the likelihood of afterdischarge (AD) termination. Second, diffuse changes in waveform cross-coherence (WxCoh), a measure of synchronization, correlated with AD termination^4^. Third, these diffuse changes were present regardless of the distance between electrode contacts^5^. Fourth, AD termination correlated with attention to, and consideration of the answer to, the posed cognitive task, rather than to the eventual spoken answer itself^6^. Finally, WxCoh responses for beta range activity decreased over the task period but then increased with AD termination^7^.

We have shown that sites where stimulation produced ADs were not necessarily those thought important for arithmetic or spelling and were not necessarily those where AST activated the brain during fMRI assessment^4^. Why should a task not related to a site where ADs occur nonetheless terminate ADs at that site? Since ADs and seizures are thought to be related^8^, the answer could lead to new and better treatments for seizures. More broadly, there is extensive literature regarding discrete regions underlying specific cognitive tasks, but we show here that there also can be brain activity that is diffuse rather than localized. Does this diffuse activity tell us about the mechanisms underlying attention to, and performance of, a cognitive task? We hypothesized, based on our previous studies, that examination of combined time and space factors would further contribute to understanding brain activity during cognitive tasks and during waking life generally.

## Methods

We have previously reported our overall methods^4^. Below we describe these, quoting from the original publication as appropriate, but with modifications made for this report. Reuse and modifications were approved by the original publisher.

### Patients

We reviewed the recordings of all patients who had intracranial electrode contacts placed for evaluation of intractable seizures and who had undergone clinical stimulation testing over 4 years. In 15 of these patients, a cognitive intervention (AST) was used in an attempt to abort ADs^3^. None of these patients reported seizures precipitated by calculation, spelling, or similar mental activities. AST was associated with AD termination in 50 trials among 12 of these 15 patients, and these are the patients reported here. For these patients, session durations ranged from 47 to 177 min; the average duration was 111 min. There were 6 males and 6 females, ages 12–53 years. (supplementary table) Clinical considerations alone determined electrode contact locations, number of contacts placed, duration of recording, number, and duration of testing sessions, contacts stimulated, and modalities tested (see below). Anticonvulsant medications varied among these patients, again based on clinical considerations alone. However, we report changes occurring over the span of a few seconds, and there were no within-patient medication changes over those brief periods. The retrospective data review for this report was approved by the Institutional Review Board of Johns Hopkins Medicine, and the requirement for informed consent was waived. All research was performed in accordance with relevant guidelines/regulations. Data were fully anonymized for the analysis. Data were accessed for this report on June 18, 2023.

### Recordings

We used platinum electrode contacts embedded in flexible silastic in linear or rectangular arrays and placed in the subdural space or implanted into the cortex (Ad-Tech Medical Instrument Corporation, Racine, Wisconsin, USA) Subdural contacts were 4 mm diameter, 1.5 mm thick subdural discs (2.3 mm exposed to the cortex, 10 mm center to center distance). Cylindrical depth electrode contacts were 1.1 mm in diameter, 1.32- or 2.41 mm in length, spaced 2.2 mm or 6.5 mm apart. Contact location with respect to underlying cortical surface anatomy was determined by direct observation in the operating room and by co-registration of pre-implantation volumetric brain MRI (1–1*.*8 mm coronal slice thickness) with post-implantation volumetric brain CT (1 mm axial slice thickness). This was determined according to anatomic fiducials using Curry (Compumedics Neuroscan, El Paso, TX, USA). Contact positions thus derived were displayed with a brain surface rendering derived from the pre-implantation MRI.

Patients underwent continuous electrocorticography (ECoG) using a Stellate Harmonie system (Natus Medical Incorporated, Pleasanton, CA 94566 USA) that could record up to 128 channels with 1000 samples per second per channel, using Schwarzer EEG Amplifiers Model 210033 (Natus Europe GmbH, Robert-Koch-Str. 1, Planegg, Germany). The video sampling rate was 30 frames per second. The machines used 16-bit A-D converters (Gain 1408, Range 4.5 Volts, Noise referred to input 1.5 Microvolts). The low-pass anti-alias filter was set to 300 Hz (20 dB/Oct Butterworth 5th Order) and high-pass to 0.0016 Hz (6 dB/Oct RC 1st Order). Analog to digital conversion was 16 bits with the Least Significant Bit (LSB) equal to 0.10 Microvolts, peak-to-peak noise of 1.5 Microvolts (equivalent to 4 bits), and Common Mode Rejection Ratio (CMRR) of 100 dB. A common average reference was used for the recordings. Since we did not know in advance where ADs might occur, we could not modify the reference for specific trials. The analyses themselves were performed between electrode contact pairs, and not between individual contacts and the reference. In all cases, recordings were continuous from all implanted contacts. The complete testing sessions were exported in European Data Format (EDF)^9^ for analysis at a later date.

### Methods of stimulation

Extraoperative functional mapping was performed using electrical stimulation via the implanted electrode contacts, gradually increasing stimulus intensity as previously described. The maximal possible stimulation intensity with our device, a Grass S12 stimulator (Astro-Med, Inc., West Warwick, RI. 02893 USA)^10,11^ was 15 mA. The charge for each pulse train was measured using a custom-built circuit and was recorded along with the ECoG. Stimulation used pairs of opposite polarity charge balanced square wave pulses, 0.3 ms in duration, repeated at 50 pulses per second. The maximal current used in these 12 patients was 11 mA (milliamperes). There was a charge of 3.6 µC (microcoulombs) per pulse. Pulse trains for the stimulation testing were 2–5 s long. Given these settings, for 2 s of stimulation there would be 100 pulses, and, at 12 mA, 360 µC would be delivered. As would be expected, since charge is linear, charge would be 180 µC at 6 mA, 120 µC at 4 mA, etc. For 4 s of stimulation, with the same parameters otherwise, 720 µC would be delivered.

When ADs occurred, BPS could be used; see below. There were 5 pulses in each train of BPS. Except for total train duration, BPS parameters were otherwise identical to those just used for the stimulation testing. As just noted, there were 3.6 µC per pulse so that, for example, at 12 mA, there would be a total of 18 µC for each BPS train of 5 pulses. As would be expected, since charge is linear, charge would be 9 µC at 6 mA, 6 µC at 4 mA, etc.

Note that charge densities produced by stimulation of electrode contacts are largely confined to the region immediately surrounding each electrode contact^11–13^. When stimulating between contacts with centers separated by 1 cm (cm) is compared with stimulating between contacts with greater separation, there is a higher current density in the region around each contact in the former case. In both cases, the current density at a contact is higher when the delivered current is higher and decreases with distance from a stimulated contact.

Stimulation intensity needed to produce functional changes varies among contact pairs, even when pairs are adjacent to one another^14–16^, and so was gradually increased for each pair tested^11^. This was done in an effort to avoid ADs and to find the minimal charge density that produced clinical changes, regardless of the distance between the electrode contacts in a pair. Stimulation methods were the same for subdural and depth contacts.

### Clinical testing

**Figure 1:**
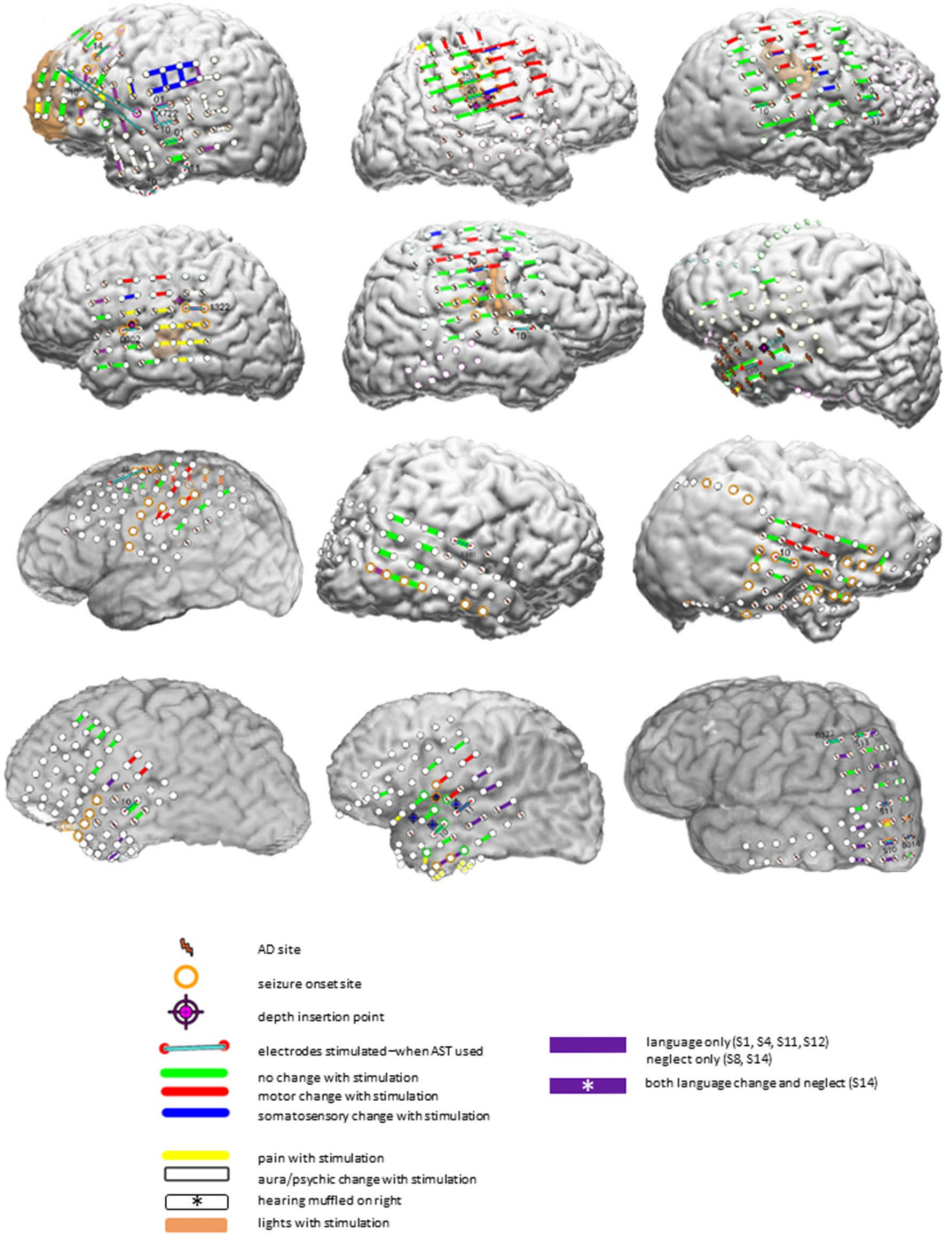
Findings on the 12 reported patients, showing functional changes during clinical testing; S1–3 in the top row, S4–6 in the second row, S7–9 in the third row, and S11,12,14 in the fourth row. In S10, 13, and 15, AST did not result in AD termination. Key: The red circles connected by blue lines indicate where arithmetic or spelling was attempted in an effort to stop ADs. Lightning bolts indicate sites with Afterdischarge (AD) spread from sites stimulated; note that ADs often cannot be seen on the stimulated channels due to “blocking”—DC baseline shift of the EEG due to the stimulation. Orange circles indicate where clinical seizures began. When seizure onset or ADs occur from depth electrodes, the same symbols are used as indicated above, but may appear near but not on the depth insertion symbol. The key indicates the type of functional change that occurred with stimulation. Note that purple was used to indicate sites with language changes, except for patients S8 and S14, where purple indicated sites where neglect occurred with stimulation, except that at three sites in patient S14, both language changes and neglect occurred with stimulation. For larger images of stimulation results, see^4^.

Patient testing occurred in their hospital rooms, with patients lying supine, with the head of the bed elevated. Patients would initially lie quietly, while stimulus intensity was slowly increased. During this, they would indicate whether they noticed any motor or sensory changes occurring in response to stimulation, and testing personnel would look for motor changes. Once stimulus intensity had been optimized for a given site without sensorimotor changes occurring, additional testing occurred. Specific tests varied and depended on clinical needs. Patients might be tested for inhibition of the ability to perform rapid repetitive alternating movements of eyes, tongue, fingers, or toes. They might be asked to perform simple non-repetitive motor acts (i.e. “close your eyes”, “raise your right hand”) or to speak, to repeat single words, or to read passages of text. They might be asked to identify pictures taken from the Boston naming test or to answer questions during testing of auditory responsive naming (i.e. where do you put food in the kitchen to keep it cold?)^17,18^. In three patients (S02, S08, S14) neglect was tested (line bisection, gap detection, shape judgment, reading left/right visual field), with neglect found during stimulation in S08 and S14. Patient S14, whose occipital lobe was tested, also reported flashing lights with stimulation. See Fig. 1 and^4^ for additional information regarding the stimulation results. We emphasize again that the choice of sites to test, and the modalities to test, were based on clinical needs alone. Also, stimulation at times can evoke pain at a particular electrode contact^19^, so that testing cannot be performed at that site. For all these reasons, not all modalities and not all electrode contacts were tested.

During testing, ongoing ECoG was monitored continuously for ADs^2,16,20^. ADs occur as abrupt changes in the ongoing ECoG, occurring after brain stimulation, but they vary in morphology^1,2,20,21^, and thresholds for evoking ADs vary from site to site and trial to trial, even when testing and retesting over short intervals^14–16^. In these patients, we found ADs of four types (1) continuous sinusoidal, (2) continuous quasi-sinusoidal appearance but spike-like morphology or sharp point at extreme of discharge, (3) repeated bursts with pause of epileptiform activity in between, (4) spike-and-slow-wave complex. Stimulation did not induce the habitual seizures for these patients^22^.

**Figure 2:**
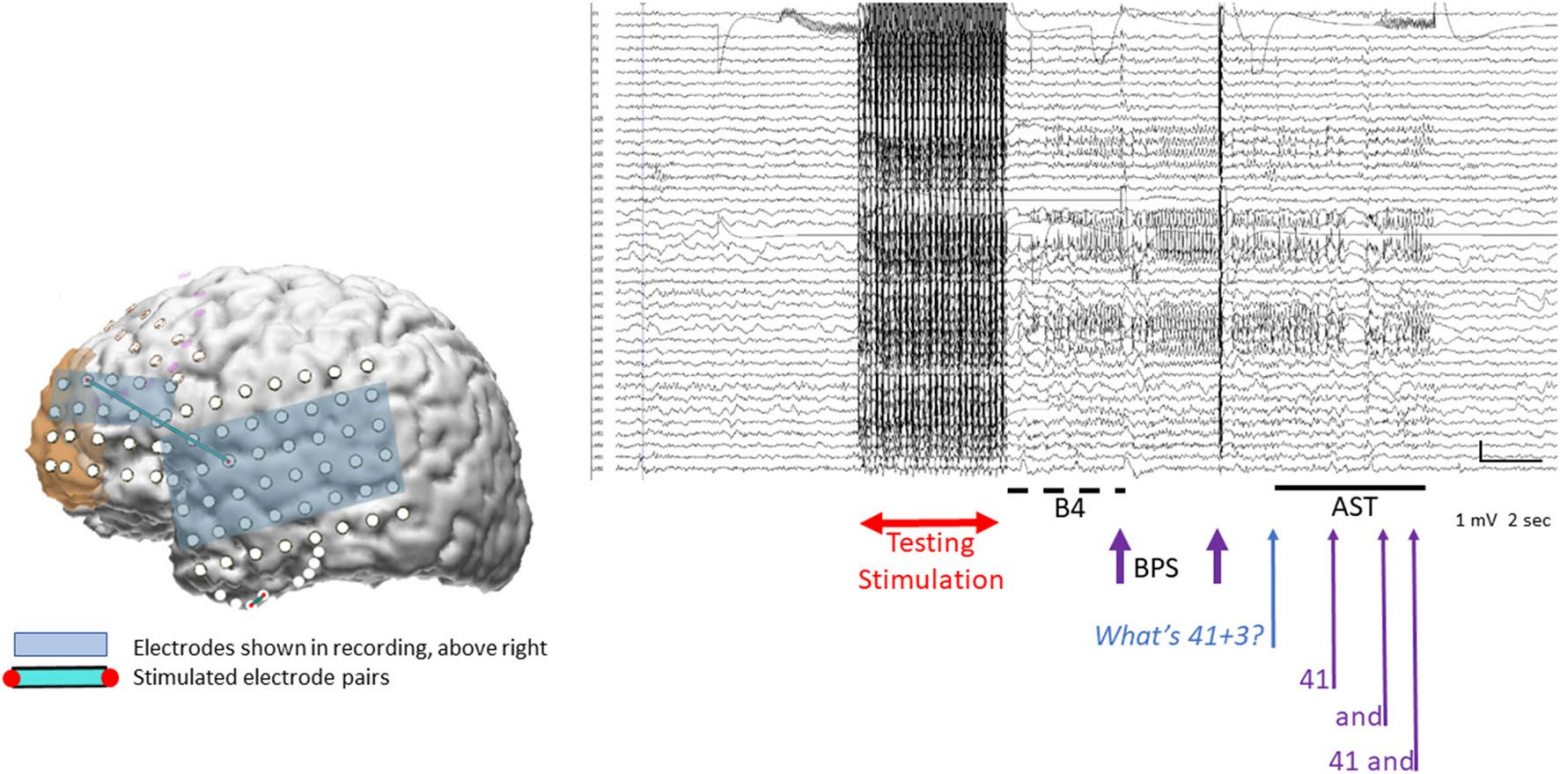
Effects on afterdischarges with stimulation and with an arithmetic question. Electrode pairs are stimulated during clinical testing (Testing Stimulation). The first segment, here called B4, occurred before an arithmetic or spelling task had been given. Additional segments occurred after the tester had begun to state the arithmetic or spelling problem. The brain image on the left shows the two pairs that were stimulated (blue lines, a longer one going from the temporal to frontal region, and a shorter one at the temporal base). The long blue line between the temporal and frontal lobes indicates where AST terminated ADs. Stimulation testing resulted in no language changes when stimulating between the frontal site and its frontal neighbor; this is indicated by the green bar between them in Fig. 1. It did result in changes when stimulating between the temporal and frontal sites. Therefore, the current at the temporal site is what interfered with language functions. This is indicated by the purple circle around the temporal electrode in Fig. 1. The ECoG recording shows results after stimulation between the temporal to frontal contact. Electrode contacts whose recordings appear on the right are highlighted with the light blue rectangular box. The horizontal double arrow indicates when clinical stimulation occurred. Next two shorter vertical purple lines indicate when brief pulses of stimulation (BPS) were administered in attempts to end the afterdischarges. Because they did not stop, an arithmetic question was asked, ‘‘What’s 41 + 3?” The onset of the question is indicated by the blue arrow. The patient answered ‘‘41... and ... 41 and”. The onset of each portion of her reply is likewise indicated by an arrow. Calibration indicates 1 millivolt and 2 s. This figure is modified from^4^ with permission.

When ADs occurred, the testing team observed the ECoG to see if ADs stopped spontaneously. Electrical stimulation can transiently saturate the EEG amplifiers after stimulation, possibly obscuring any ADs that might be present, a phenomenon called blocking. Because of this, testing personnel waited at least two seconds to ascertain whether ADs continued^2^. If ADs did not stop, an effort was made to terminate them, with BPS given at the same electrode contact pair where stimulation had produced the ADs^2^, or the patient might be asked to solve arithmetic problems (i.e. what is 27 + 14, what is 73–76, or (patient S1) count backwards, or asked to spell words backwards, or (patient S1) to recite the alphabet backwards. ADs vary considerably with respect to when they occur and how long they last. Because of this, once ADs were noted, the length of time to wait before using BPS or AST was a clinical decision on the part of the testing team. There were no specific instructions regarding whether to use BPS or AST or regarding what task to present to the patient when AST was used (Fig. 2).

Some trials where the cognitive task was delivered could also contain several closely spaced BPS. In such cases, it was not possible to obtain clear BPS-free sections for analysis. On the other hand, some ADs continued for several seconds and did not respond to the initial AST. This allowed more than one cognitive task. In these cases, the B4 segment was selected after the AD had started but before the first cognitive task question and answer. The first AST segment samples were taken from the ECoG after this. There was a pause after which a second cognitive task was given to the patient. Second AST segments were then obtained. The first arithmetic or spelling task section was scored as a failure. The second section could be either scored failed or successful depending on whether the ADs stopped within 2 s^2^. We only selected trials where we could obtain periods for both the B4 and AST segments that did not contain any brief pulses of stimulation.

The patients were likely aware that ADs were occurring because of the reactions of the testing personnel, but they were given the cognitive tasks without any other specific warning. Patients recognized all the numbers and words they were asked. Tasks were used during testing in either hemisphere, were used whether or not ADs were in a region that literature has suggested might be important for arithmetic or spelling, and were used whether or not the AST had been preceded by BPS^17,23–28^. Electrode contacts stimulated, intensities used, changes produced by stimulation, the occurrence of ADs, and effects of interventions (brief pulses of stimulation, arithmetic, spelling) were recorded in real-time using a locally developed (RPL) program, called Report.

**Figure 3:**
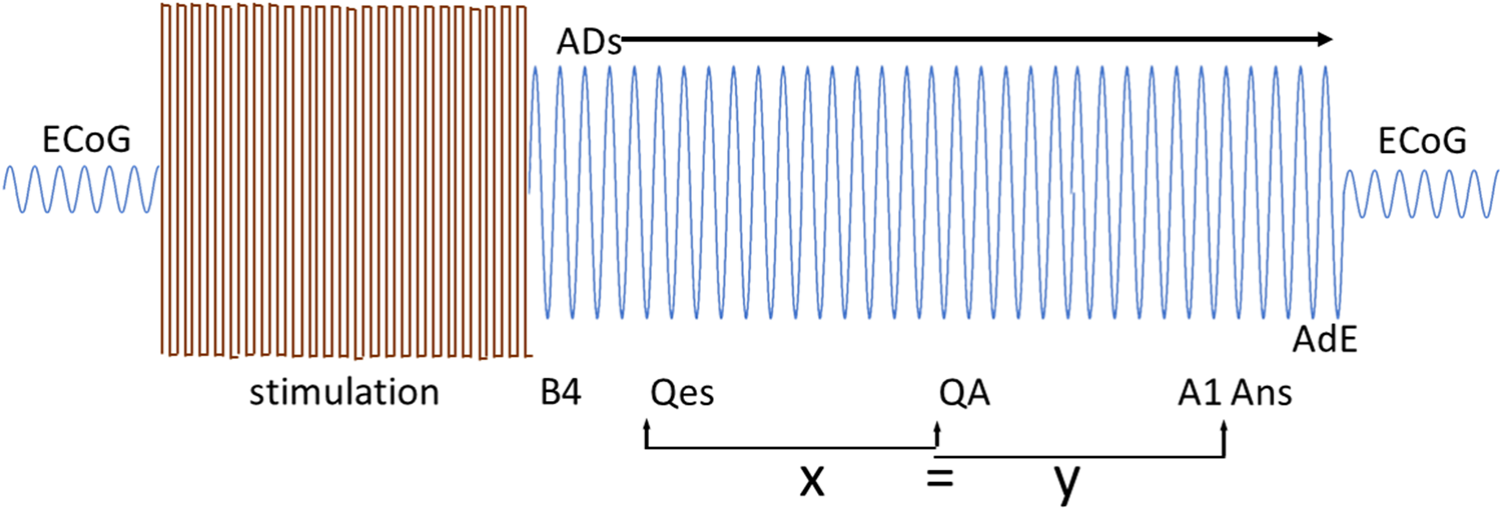
Schematic of the relationships between the time segments. Ongoing electrocorticographic brain activity (ECoG) is interrupted by brain stimulation which in turn produces afterdischarges (ADs). A baseline segment is defined (B4, see Methods). Next is a segment beginning when the arithmetic or spelling task is initiated, which is labeled as Qes in the figure. Another segment begins when the patient begins to answer (Ans). Between these two segments is QA. The time from the beginning of Qes to the beginning of QA is equal to the time between the beginning of QA and the beginning of Ans. Just before Ans is A1; this segment ends when Ans begins. The AdE segment ends when the ADs end. Each segment lasts one second.

We analyzed several one-second segments of the ECoG recorded during the task^7^ (Fig. 3). The first segment, called Qes, began when the AST question was initiated by the testing personnel. Another (Ans) began when the patient began a verbal answer. A third (QA) began halfway between when the question (Qes) began and when the answer (Ans) began. A fourth (A1) began one second before the beginning of the patient’s verbal answer. The fifth (AdE) began one second before the end of the ADs. We compared these segments to a baseline which we called B4 and which occurred before the beginning of the question. To obtain this baseline, we began with the 4-s baseline described in previous papers^4,5^. We divided the 4 s into four consecutive one-second segments and averaged these, with a resulting 1-s baseline.

We also separated the recordings for each of these segments into five additional subgroups^5^. The first subgroup, 10% of the total, consisted of electrode contacts whose centers were 1.9 cm or less from one another, i.e., the eight nearest neighbors. The other subgroups, each constituting 22.5% of the remaining pairs, had distances between contact centers as follows: 1.9 cm–< 3.4 cm, 3.4 cm–< 5.1 cm, 5.1 cm–< 7.3 cm, 7.3 cm or more. We emphasize that we studied WxCoh between a given contact and each of the other contacts that had been implanted and from which satisfactory recordings could be obtained, as summarized above and in our previous reports. For example, if ADs occurred when stimulating between two electrode contacts in the frontal lobe, distances evaluated were not only with respect to each of those two contacts but also with respect to distances relative to every other implanted contact, regardless of the contact location.

After dividing the data into the five time and five distance groups, there were 25 subgroups. It was these that we analyzed for this study, assessing WxCoh at each time/distance segment relative to that of the baseline.

### Analysis of electrocorticography

We previously evaluated a total of 116 trials in 15 patients, with a total of 1269 electrode contacts and 54,912 electrode contact combinations^4^. However, as described above, AST was associated with AD termination in 50 trials among 12 of these patients, and we studied these 50 trials for this report. In these 12 patients, there were 930 subdural and 40 depth electrode contacts. Among the total of 970 contacts, there were 39,871 combinations. This included all possible combinations of 2 electrode contacts among the monitored contacts, where the order of the 2 contacts did not matter, i.e. combinations not permutations. For example, for 4 electrode contacts, called A, B, C, and D, the possible combinations are AB, AC, AD, BC, BD, and CD. The absolute polarity of the waveform recorded from A to B would differ from the polarity of the waveform recorded from B to A. However, the results of coherence calculations, such as those we performed, would be the same.

Analysis of the records was done using two programs, VZ7.exe and WCC5.exe, both locally developed (WRSW) programs written in ‘C’. To view ECoG we used VZ7.exe, which allowed us to review and measure both the ECoG and video at any desired level of accuracy, down to the millisecond. VZ7.exe contains calculation modes within it and allows export to MATLAB (MathWorks, Natick, MA 01760 USA). VZ7.exe allowed marking EDF files with the times of the baseline and task sections to be analyzed. The times of the cognitive task questions and answers were also marked with VZ7.exe so that positions of all the relevant events in each trial could be seen at the same time on one screen. To assess the degree of synchrony in the ECoG, we calculated mean wavelet cross-coherence (see below) between all pairs of implanted electrode contacts using WCC5.exe. WCC5.exe uses the events marked with VZ7.exe to locate the sections of the ECoG where wavelet cross-coherence measurements are made.

Brain rhythms traditionally are divided into several groups, for example, delta (below 4 Hz), theta (4–7 Hz), alpha (8–13 Hz), beta 14–40 Hz), and gamma (above 40 Hz)^29^. There are differences between authors regarding the precise divisions between frequencies (see for example the Wikipedia entries for these). Although we use these terms in the general sense in this report, we used wavelet scales for our actual calculations.

We calculated WxCoh for all electrode contact pairs over a range of wavelet scales with peak frequencies from 7.13 to 40.05 Hz. These in our previous papers had been significantly associated with AD termination^5–7^, and spectral analysis had shown these to contain the majority of the WxCoh changes^7^. For these calculations, a scale can be thought of as a surrogate for frequency. As indicated above, the data were analyzed for two 1000 ms windows containing ADs. The lengths of time for the questions to be asked and answered were such that ADs were present in all data segments analyzed, and no brief pulses of stimulation were present.

### Wavelet cross-coherence

The hypothesis is that when a cognitive task stops an AD it does so by disrupting the activity that sustains the AD. We are testing whether the changes can be reflected by a change in the coherence of the electrical activity at some frequency. We measured coherence in all combinations of pairs of the recorded electrode contacts.

Initially, we used a wavelet cross-correlation function^30^ but found, as have others^31^, that in some cases the calculation failed to produce a peak in the correlation function, and a peak is needed to determine a cross-correlation value. This is the risk in using cross-correlation on short data sequences^31^.

Coherence is a similar measure of the relationship between signals that is the frequency domain equivalent of correlation in the time domain and has been used by several investigators to study seizure location and spread in intracranial recordings^32–34^. The wavelet cross-coherence function devised here always produces a result because cross-coherence functions do not need to produce a peak. Studies have found wavelet coherence to be equivalent^35^ or superior^36^ to Fourier-based methods of measuring coherence. In addition, wavelet coherence can be used to study single-trial brain signals^37^. The addition of wavelets localizes the measurements in both time and frequency^38^.

Encouraged by these previous results, we devised a simple method to calculate wavelet cross-coherence. This wavelet cross-coherence measure uses a wavelet formed by taking a fixed number of cycles of a sine and cosine wave to form a complex wave and then applying a Gaussian-like exponential envelope to it. This then is a variant of the Morlet wavelet. The scale of this wavelet is then dilated while keeping the number of cycles the same to form a lower-frequency version. This process is repeated several times to produce a family of discreet wavelet scales that cover the whole frequency range for this data set. The step from one wavelet scale to the next is chosen with some overlap in frequency so that the whole frequency range is fully covered. The steps, in decreasing frequency, are given by:

1 $${F}_{n+1}={F}_{n} \left({C}_{y}- {C}_{s}\right)/{C}_{y}$$

where:

${{F}_{n}, F}_{n+1}$ are the current and next wavelet frequency scales. The interval between frequencies is a constant ratio, as explained in the next few sentences.

${C}_{y}$ is the number of cycles in the wavelet family. In this study it is set to 6.

${C}_{s}$ sets the spacing of the wavelets in scale, which sets the duration of the wavelet, and the difference in frequencies covered when moving between scales. Set to 1.5, this can be lowered to improve frequency coverage by making the wavelets closer in frequency.

With ${C}_{s}$ = 1.5 and ${C}_{y}$ = 6, the ratio between consecutive wavelet frequencies is 0.75.

In this study, we have a total of 7 separate wavelets, each of which assesses a different range of frequencies.

As ${C}_{s}$ is increased, the number of wavelets within the family of wavelets that would assess the total range of frequencies would be reduced after calculation of Eq. (1). The risk in doing this is that each of the separate wavelets could assess a narrower range of frequencies so that some frequencies between two wavelets might not be appropriately assessed by either wavelet. (See next paragraph.)

**Figure 4:**
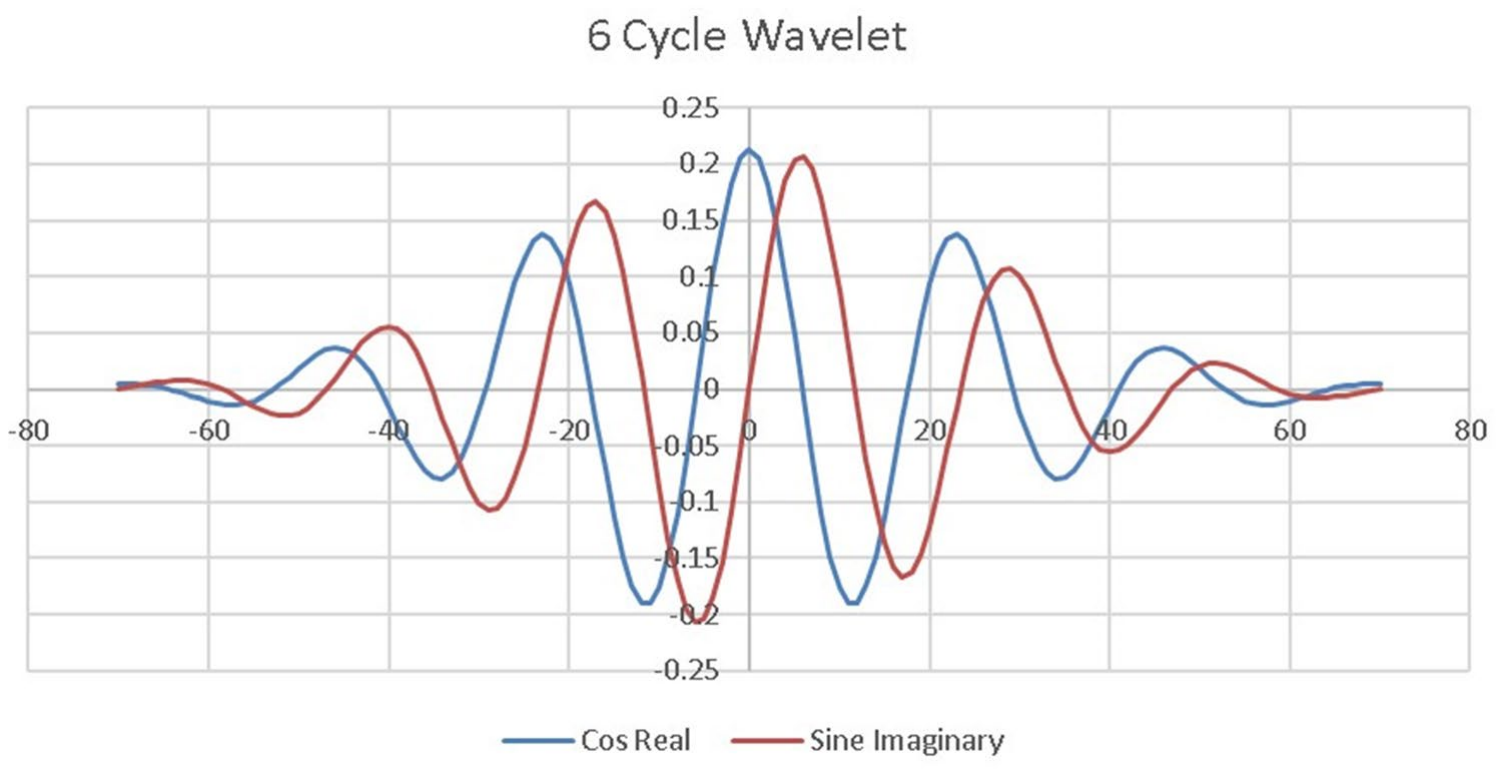
Example wavelet. See text for discussion. This figure is from^4^ with permission.

**Figure 5:**
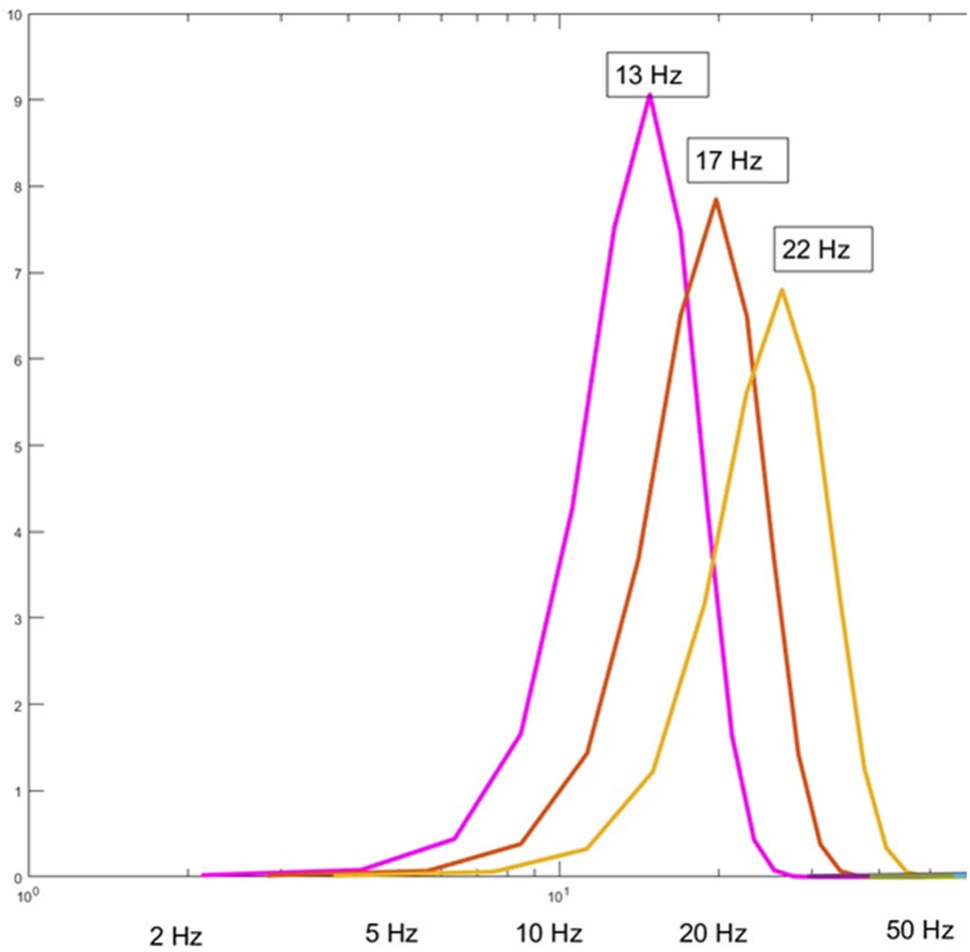
Example of overlap between adjacent wavelets showing that the frequency space is covered continuously without gaps. The Y axis is in arbitrary units but reflects the sensitivity (i.e. frequency response) at the given point. This figure is modified from^4^ with permission.

The wavelet used consists of 6 cycles of a complex wave i.e. it has real (cosine) and imaginary (sine) parts (see Fig. 4). In this study, we set ${C}_{s}=1.5$ in Eq. (2) below. This then covers the frequency range from 7.13 to 40.05 Hz in 7 wavelets in a logarithmic fashion such that the ratio of the frequency of adjacent wavelets is 1.33 going from low to high. The lowest frequency wavelet for this study is 7.13 Hz because this is the lowest frequency for this family of wavelets for which six cycles would fit into the one-second segments we studied. The correlation between adjacent wavelets is 0.75 because there are no gaps in frequency space in the range of this study i.e. the coverage is continuous from 7.13 to 40.05 Hz (Fig. 5).

Although ADs do not appear visually to be simple sine waves, the basis of the wavelet used here is a sine wave. However, by changing ${C}_{y}$ we can explore different wavelets. For example, larger values of ${C}_{y}$ will produce wavelet families that are narrower in frequencies for which they are sensitive, but which will require a wider time window to differentiate a given frequency from a nearby frequency. The complex wavelet is then given by:

2 $${W}_{un}\left(Fn, t\right)={ e}^{-k{t}^{2}}( {\text{cos}}\left(2\pi {F}_{n}t\right) + j sin\left(2\pi {F}_{n}t\right) )$$

2.1 $$W\left(Fn, t\right)= \frac{{W}_{un}\left(Fn, t\right)}{\sqrt{\frac{\sum_{\tau ={-W}_{len}}^{\tau =+{W}_{len}}{\left|{W}_{un}({F}_{n},\tau )\right|}^{2}}{2}}}$$

where:

$$j= \sqrt{-1}$$

${F}_{n}$ is the frequency of the chosen wavelet derived from Eq. (1).

$k$ is the taper for the Gaussian-like exponential envelope and is chosen so that the amplitude of the wavelet at each end is 1.8% of the center amplitude. For $k=1$ this is the Morlet wavelet.

$t$ is the time index of the wavelet, with t = 0 in the middle of the wavelet. It runs from ${-W}_{len}$ to $+{W}_{len}$

${-W}_{len}$, $+{W}_{len}$ are the limits of the wavelet ${e}^{-k{t}^{2}}$ is the Gaussian-like envelope applied to the original waveform with k indicating the taper and t indicating the point along the wavelet.

${W}_{un}\left(Fn, t\right)$ is the un-normalized wavelet which is then normalized by dividing it by its root mean square (RMS) as shown in Eq. (2.1).

$W({F}_{n,t})$ is the normalized wavelet used for the cross-coherence calculation.

For given wavelet $Fn$ and a pair of electrode contacts $X$ and $Y$, we take a fixed length sample of data $X\left(t\right)$ and $Y\left(t\right)$. Each of these samples of data is convolved with the wavelet $W\left(Fn,t\right).$

3 $${W}_{X}\left({F}_{n,}t\right)= \sum_{\tau ={-W}_{len}}^{\tau =+{W}_{len}}X\left(t-\tau \right)W({F}_{n},\tau )$$

3.1 $${W}_{Y}\left({F}_{n,}t\right)= \sum_{\tau ={-W}_{len}}^{\tau =+{W}_{len}}Y\left(t-\tau \right)W({F}_{n},\tau )$$

The wavelet cross-coherence value that we use is then given by the dot product of ${W}_{X}\left({F}_{n},t\right)$ and the conjugate of the same function for the other contact, ${*W}_{Y}\left({F}_{n},t\right),$ and normalized by the amplitude of each sample of data $X\left(t\right)$ and $Y\left(t\right)$.

4 $${WXCoh}_{X*Y}\left({F}_{n},t\right)= \frac{\sqrt{\sum_{\tau =-{W}_{len}}^{\tau =+{W}_{len}}{\left|{W}_{X}\left((t-\tau ),{F}_{n}\right)*{W}_{y}((t-\tau ),{F}_{n})\right|}^{2}}}{\sqrt{\sum_{\tau =-{W}_{len}}^{\tau =+{W}_{len}}{\left|{W}_{X}\left((t-\tau ),{F}_{n}\right)\right|}^{2}}\sqrt{\sum_{\tau =-{W}_{len}}^{\tau =+{W}_{len}}{\left|{W}_{Y}\left((t-\tau ),{F}_{n}\right)\right|}^{2}}}$$

We used WCC5.exe (see above) to compute Eq. (4) for all scales for a selected sample of data at any given time point in an ECoG file saved in EDF. WCC5.exe allows the constants in Eqs. (1) and (2) to be changed as desired so that different wavelets can be studied. Since the wavelet frequencies overlap to some extent, there are no gaps in coverage in the frequency ranges used in this study. Wavelet cross-coherence values are always in the range 0.0 to 1.0 due to the normalization denominator term in Eq. (4)

As noted above, the clinical stimulations sometimes cause ADs which were then treated by the clinical team with brief pulse stimulation^2^ and/or a cognitive task in an attempt to stop the ADs. Wavelet cross-coherence was calculated for two short sections of ECoG during these ADs. The first section, referred to as B4, was taken after the AD had started but before the cognitive task was given. As also described above, the cognitive task consisted of patients being asked to answer an arithmetic or spelling question. The succeeding sections were taken while the patient thought about and answered the question. As in our previous paper^2^, if the ADs stopped during or within 2 s after the end of the cognitive task, without further intervention, it was treated as a success. If ADs continued for more than two seconds after the end of the answer, the trial was treated as failed. ADs were continuously present in the B4 sections, and the B4 section ended before the question for the cognitive task started. Arithmetic or spelling task sections occurred after the end of the B4 sections and always were initiated while ADs were present.

The first step to computing wavelet cross-coherence was to apply Eq. (2) to the 1 s of each member of a given pair of electrode contacts. Next Eq. (4) was computed for these two 1-s periods to produce the value of wavelet cross-coherence for each pair of electrode contacts. This process was repeated for all pairs for each B4 and AST segment.

### Statistics

The primary analysis compared changes across the five distance groups among all inter-contact pairs, separately for each frequency and time segment. For each patient, trial, time segment, distance group, and frequency, we calculated the mean change in wavelet cross-coherence compared to the B4 baseline over all inter-contact pairs. We used logistic regression to compare the WxCoh changes by time segment relative to the B4 baseline across the five distance groups, employing generalized estimating equations with a working independence covariance matrix to account for correlation among multiple trials within a patient^39^. We fit separate models by frequency since our previous study of AST in these patients^4^ found a significant interaction between WxCoh and frequency on the probability of AD termination. Because of their similarity (see below), we also combined the middle three distance groups and carried out exploratory analysis on the resulting close, middle, and far distance groups to increase statistical power for detecting differences. Tests were two-sided with an alpha of 0.05. SAS 9.4 was used for the analysis.

## Results

Stimulation usually was between electrode contacts that were adjacent to one another; Fig. 1 shows exceptions. For the 12 patients, 43 of the stimulated contact pairs had a center-to-center separation of 1 cm. For 3 pairs, the separation was 3.2 cm, and, for 4 pairs, the separation was 6.7 cm or more.

**Figure 6:**
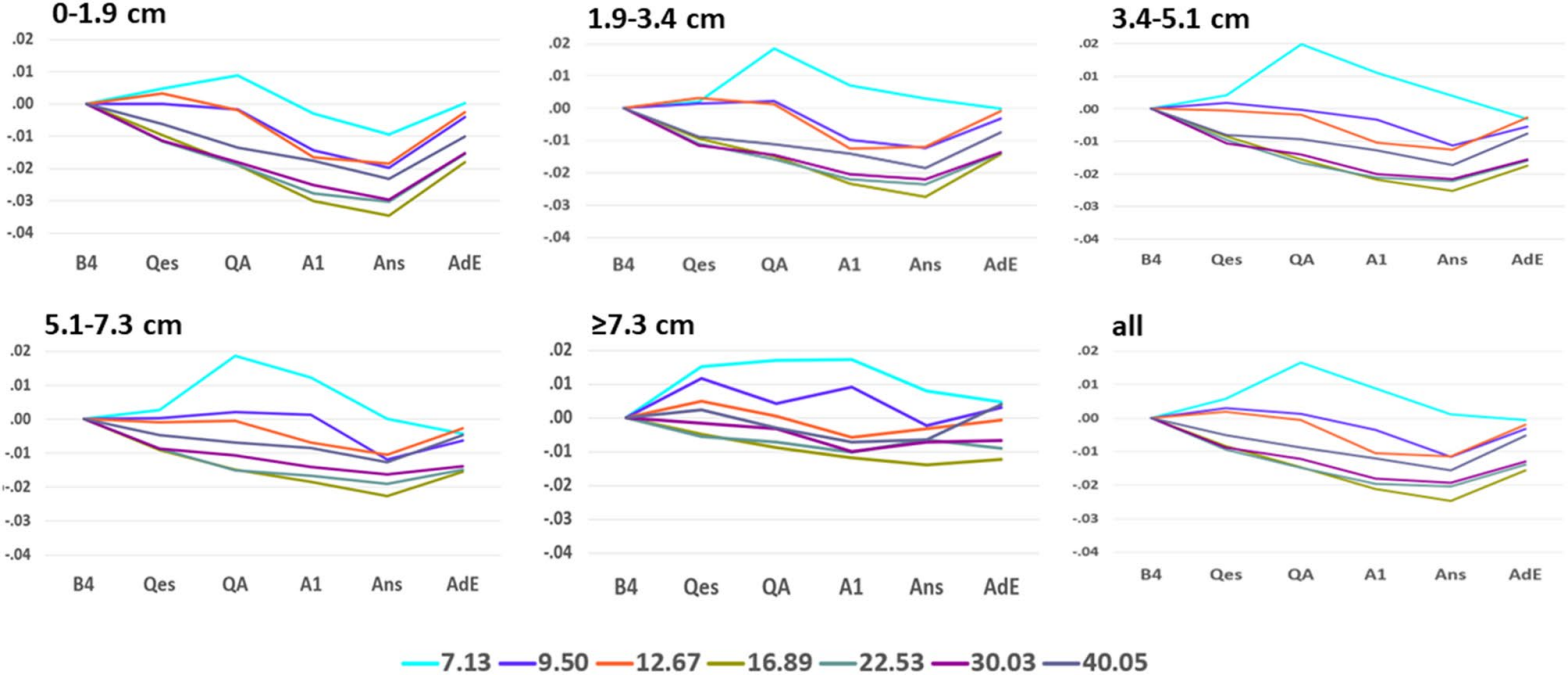
Mean change in WxCoh by distance group. The figure plots the mean changes in WxCoh by time segment separately for each of the five distance groups. The label over each figure indicates the inter-electrode distances included in that plot. The bottom right figure (all) shows the results for all inter-contact distance pairs. This is the same data as Fig. 3 in our previous paper^7^, but with results placed on the same y-axis as the other items in this figure. The five distance groups are 0–1.9 cm, 1.9 cm–< 3.4 cm, 3.4 cm–< 5.1 cm, 5.1–< 7.3 cm, 7.3 cm and above. The x-axis indicates the time segment; see “Methods”. The y-axis indicates the mean change in WxCoh during that time segment with respect to the B4 baseline. The lines indicate the individual wavelet scales, expressed as each scale’s peak frequency in Hertz (Hz). See the video for an animation of these changes.

Separately for each of the five inter-contact distance groups, we plotted the mean WxCoh changes from B4 baseline by time segment and frequency (Fig. 6). For the four closest distance groups, WxCoh in the 9.5–40.05 Hz frequency ranges either progressively decreased, or slightly increased and then decreased until the Ans segment. Responses then increased for the AdE segment. While not parallel, the directions and amounts of change from one time segment to the next were similar, particularly for the five higher frequencies. This pattern of similar changes for these frequencies was most evident for the closest inter-contact distances and less evident as distances increased. The 7.13 Hz responses increased for several segments and then decreased, except for an increase for the AdE segment with respect to the closest distance group (0–1.9 cm). The overall appearances for the three middle distance groups (1.9–3.4, 3.4–5.1, 5.1–7.3 cm) were quite similar to one another, and also similar to the responses for all contact pairs (all) (also see^7^).

**Figure 7:**
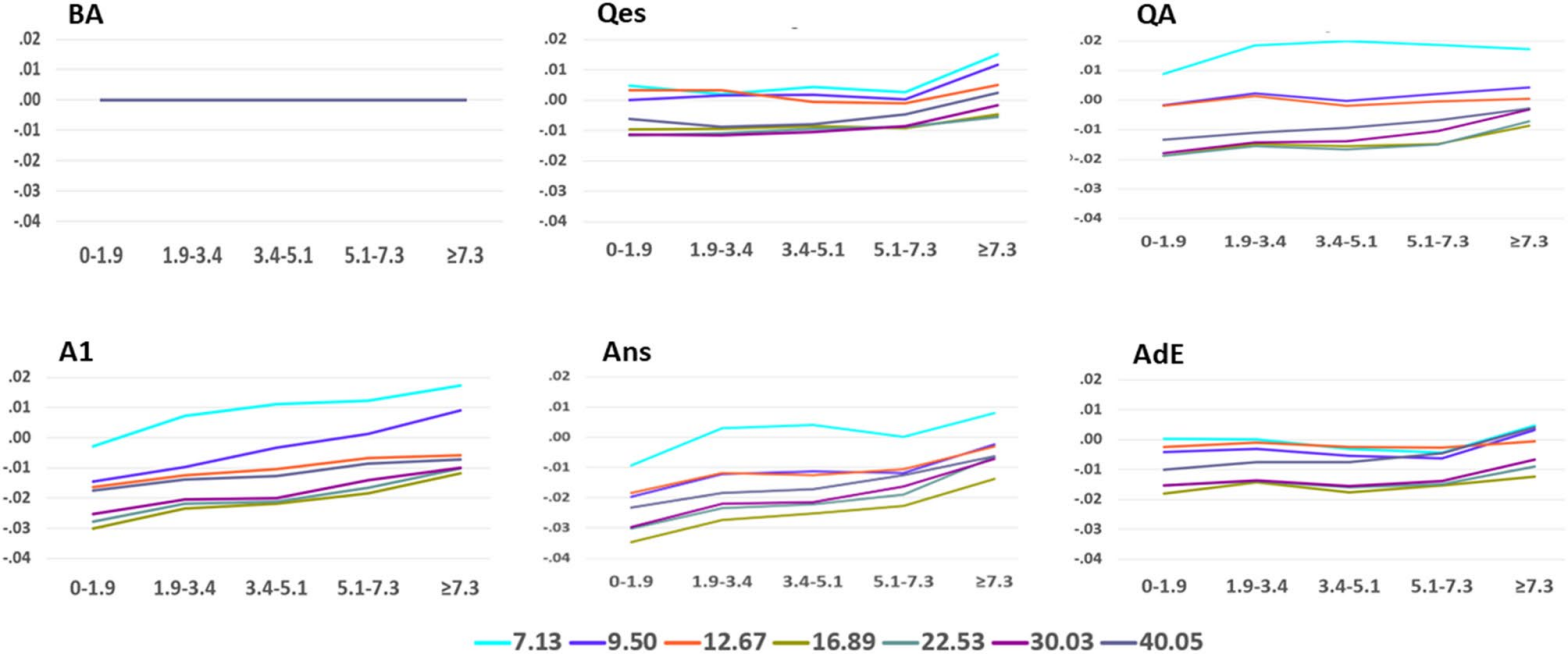
Mean changes in WxCoh by time segment. The figure summarizes the same information as in Fig. 6 but reformatted to compare WxCoh changes for the five time segments, with respect to the five inter-contact distance groups. The less the y-axis point-to-point differences for a line, the more similar the results are for the respective inter-contact pair distances. The flat line during the BA baseline is meant to indicate that subsequent results are shown as changes with respect to the baseline. When the lines show similar slopes, this suggests the possibility of similar changes underlying the changes in WxCoh results. The title above each segment of the figure indicates the time segment, see “Methods”. The x-axis indicates the inter-contact distance group in cm, and the y-axis the mean change in WxCoh with respect to the B4 baseline. The lines indicate the individual wavelet scales, expressed as each scale’s peak frequency in Hertz (Hz). See the video for an animation of these changes.

We also evaluated mean changes in WxCoh from B4 baseline for each of the five time segments plotting this for frequency with respect to the five inter-contact distance groups **(**Fig. 7). The less the y-axis point-to-point differences for a line, the more similar the WxCoh results are for the respective inter-contact pair distances. When lines show similar slopes, this suggests the possibility of similar changes between the connected WxCoh result points. WxCoh values during the Qes segment are similar for the lowest three and also for the highest four frequencies for the closest four distance groups. Values in general increase in the ≥ 7.3 cm distance. During the QA segment, there are clusters involving five of the seven frequencies with separation of the 7.13 Hz results. During the A1 segment, there is a progressive increase in WxCoh for all seven frequencies; this is less marked for two (12.67 Hz, 40.05 Hz). The 7.13 Hz values continue to be higher than the others. During the Ans segment, WxCoh changes for each frequency increase between the first and second distance groups, then WxCoh changes are relatively constant for the lower frequencies and increase slightly for the higher frequencies for the middle three distance groups (i.e., second, third, and fourth distance groups). Then all changes increase between the fourth and fifth distance groups. WxCoh changes during the AdE segment tended to increase slightly between the first and second distance groups, were similar for the middle three distance groups, and then increased between the fourth and final distance groups.

We found no significant differences in the overall tests comparing changes across the five distance groups by time segment and frequency (Table 1). To further evaluate the differences due to distance, we grouped the results for the middle three distance groups and performed the same calculations, comparing changes across the three resulting distance groups (close, middle, and far) for each time segment and frequency. Overall tests showed significant differences during the A1 and Ans segments, but not during the other segments. (Table 2) Exploratory pairwise comparisons after this regrouping showed differences among several time segments, particularly during the A1 and Ans segments (Table 3).

**Table 1:** Overall test of significance, comparing WxCoh changes across five distance groups, for each frequency and time segment, adjusting for correlations within patients.

Time segment	Frequency (Hz)	Chi-square test	p-value
Qes	7.13	4.57	0.33
Qes	9.50	6.40	0.17
Qes	12.67	7.24	0.12
Qes	16.89	6.41	0.17
Qes	22.53	3.43	0.49
Qes	30.03	4.67	0.32
Qes	40.05	5.00	0.29
QA	7.13	3.90	0.42
QA	9.50	5.75	0.22
QA	12.67	2.68	0.61
QA	16.89	5.96	0.20
QA	22.53	6.29	0.18
QA	30.03	6.18	0.19
QA	40.05	4.67	0.32
A1	7.13	4.66	0.32
A1	9.50	7.00	0.14
A1	12.67	6.53	0.16
A1	16.89	6.99	0.14
A1	22.53	5.58	0.23
A1	30.03	8.14	0.087
A1	40.05	7.77	0.10
Ans	7.13	4.09	0.39
Ans	9.50	5.50	0.24
Ans	12.67	7.52	0.11
Ans	16.89	6.90	0.14
Ans	22.53	7.12	0.13
Ans	30.03	7.13	0.13
Ans	40.05	7.69	0.10
AdE	7.13	2.12	0.71
AdE	9.50	6.66	0.16
AdE	12.67	5.49	0.24
AdE	16.89	5.29	0.26
AdE	22.53	7.73	0.10
AdE	30.03	7.83	0.098
AdE	40.05	5.71	0.22

Each chi-square test has 4 degrees of freedom. None of the overall tests were significant. Time segment indicates the time segment of the sample, see “Methods”. Frequency (Hz) indicates peak frequency in Hz for the respective wavelet scale. The Chi-Square Test indicates the value of the chi-square test statistic. The p-value is based on a chi-square test with 4 degrees of freedom.

**Table 2:**
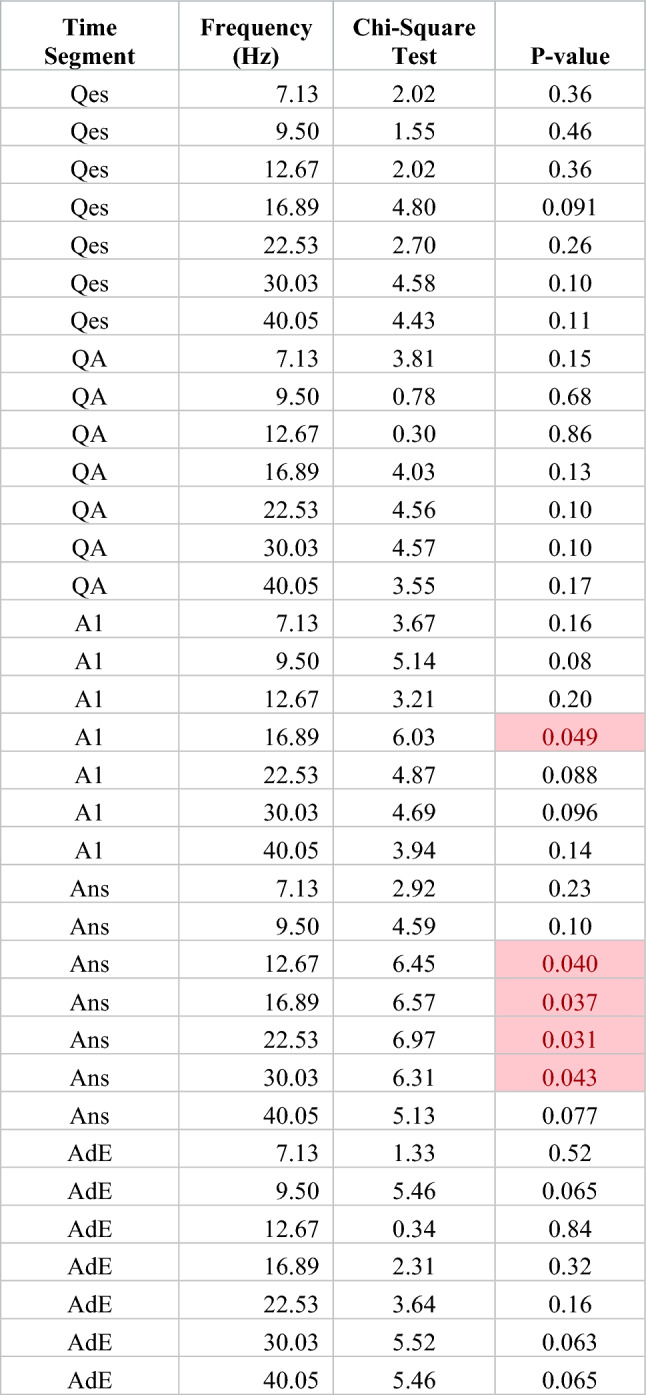
Overall tests of significance, comparing WxCoh changes across three distance groups (after combining the middle three distance groups, see “Methods”), for each frequency and time segment, adjusting for correlation within patients.

Each chi-square test has 2 degrees of freedom. Time segment indicates the time segment of the sample, see “Methods”. Frequency (Hz) indicates peak frequency in Hz for the respective wavelet scale. The Chi-Square Test indicates the value of the chi-square test statistic. The p-value is based on a chi-square test with 2 degrees of freedom. Highlighted cells indicate p-values < 0.05.

**Table 3:**
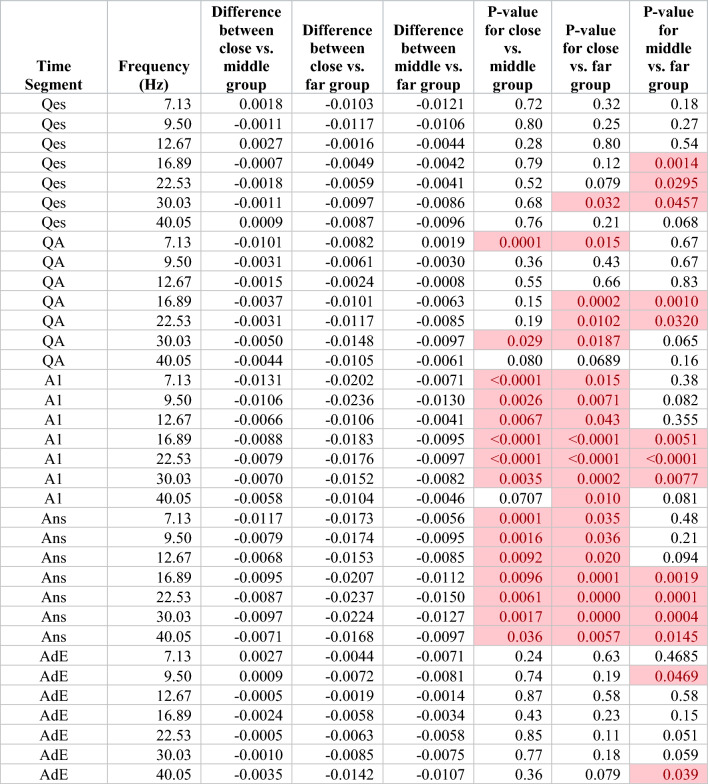
Pairwise tests comparing WxCoh changes among the three inter-electrode contact distance groups (after combining the middle three distance groups, see “Methods”), for each frequency and time segment, adjusting for correlation among multiple trials within patients.

Time segment indicates the time of the sample. For time segment abbreviations, see methods. Frequency (Hz) indicates peak frequency in Hz for the respective wavelet scale. Differences are the differences in mean WxCoh changes between the two distance groups being compared. In the last three columns, close includes the 0–1.9 cm results, middle the 1.9–3.4, 3.4–5.1, and 5.1–7.3 cm results, and far the ≥ 7.3 cm results. Highlighted cells indicate p-values < 0.05.

## Discussion

### Non-pharmacologic methods for terminating seizures

This report is an outgrowth of our interest in the occurrence of ADs in patients undergoing cortical stimulation. This occurred during evaluations to determine if patients were candidates for surgery to control their intractable seizures. For some such patients, electrode contacts are placed on and in the brain for two reasons. The first is to try and locate the site or sites of seizure onset. The second is to determine regions importantly involved in movement, somatic sensation, vision, language, and other functions, and this determination is done in part by brain stimulation. One possible side effect is that stimulation can produce ADs, which interfere with function localization and can evolve into epileptic seizures. These seizures often do not originate in the sites where clinical seizures originate and interfere with the seizure evaluation.

Region-specific tasks have been used since at least the second century to control seizures. Two examples are tying a ligature around the arm^40^ or clenching the fist^41^ to terminate seizures beginning with sensations in the hand. Similarly, Efron reported that an olfactory stimulus could abort a seizure which included olfactory hallucinations^42,43^. Since region-specific tasks can terminate seizures, they likely could also terminate ADs. We suggest this occurs because the region-specific task activates a network in the same region as that participating in the AD. The co-localization of the two disrupts the ADs^3^. However, in both Gower’s and Efron’s cases, seizure inhibition eventually could occur without the original region-specific terminating stimulus. Efron suggested that seizure arrest in his patient was likely due to “activation of a widespread inhibitory system” and that seizure arrest in Gower’s patient was similarly due to “a conditioned, non-specific stimulus”^42^. In addition, in Efron’s patient, the olfactory stimulus was later linked to the presentation of a bracelet. He reported that “the thought of the bracelet was later sufficient to arrest a seizure”^42^. Subsequently, Wolf emphasized that non-specific interventions could result in improved seizure control^41^. Considerations of this kind led us to try the methods of AD termination reported here.

### Cognitive tasks and afterdischarge termination

As summarized above, a variety of cognitive activities can abort epileptiform activity^1,41,42^ and, in keeping with these observations, we found that cognitive effort—asking patients to perform an arithmetic or spelling task (AST)—often caused the ADs to end^44^.

We initially studied the coherence of 70–110 Hz activity during the AST using network analysis. We found that, when ADs were primarily located within a single stable brain community, the AST was more likely to result in the end of the ADs^3^.

We next used WxCoh to study the AST in these patients, using 19 wavelet scales with peak frequencies between 1.69 and 300 Hz^4^. Examining this broader range of frequencies, we found decreased coherence in the 7.13–22.53 Hz frequency ranges.

To understand this further, we next separated the inter-contact combinations by inter-contact distance^5^. Coherence decreases continued to be found, regardless of the distance between electrode contacts in a pair. We emphasize again that the distances assessed were not distances relative to locations where stimulation had just resulted in ADs. Rather, these were distances throughout the cortex. For example, if a pair of electrode contacts, one in the frontal pole and one in the Rolandic area, were a particular distance apart, and another pair, both in the temporal lobe, were the same distance apart, they would be in the same distance group, regardless of where stimulation might have resulted in ADs. Therefore, the distance effect we found is a property of interactions throughout the cortex.

Next, we separated the ECoG into five time segments occurring within the AST trial period^7^. We found progressive and significant coherence decreases over time for the 16.89, 22.53, and 30.03 Hz frequency groups, with coherence increasing with AD termination. Coherence first increased and then decreased for the 7.13 Hz frequency range.

Based on these results, we now divided the inter-contact coherence groups into 25 groups, combining the five distance groups^5^ and the five time segment groups^7^. Overall, there were no significant differences between contact combinations by distance, regardless of time segment, but when we grouped the combinations by both time segment group and distance group, there were significant differences during the A1 and Ans time segments. The results for the three intermediate inter-contact distance groups were similar to one another, so we combined these into one group and after the reduction from five to three groups, exploratory analysis showed additional differences.

In summary, this study continues to show that there are WxCoh changes throughout the cortex during the AST but also shows that there are gradations in these changes, with the effects most marked during particular inter-contact distances and time segments. Changes in WxCoh values among the time and distance groups were not the same but were often quite similar. This suggests that there may be similar and widespread underlying mechanisms, but there are gradations of these effects, depending on the portion of the task and the electrode contact distance group assessed. These pancortical changes coexist with the localized network changes we previously reported^3^.

Coherence is a measure of synchronization. Previous reports have concluded that *increased* synchronization of activity among cortical regions is associated with *poorer seizure control* in general and poorer responses to epilepsy surgery in particular^45–48^. Consistent with this, we found that *decreased* coherence was associated with *improved AD control*^4^.

Arithmetic and spelling functions have been localized to regions in the temporal, parietal, and frontal lobes and the thalamus^17,23–28,49–51^. In several of our patients, as well as in controls, we studied distributions of activation on fMRI in response to AST. Areas activated included the frontal operculum, dorsolateral prefrontal cortex, anterior cingulate cortex, lateral parietal cortex, (primarily anterior) insula, temporal occipital cortex, and basal ganglia (primarily putamen)^4^. However, although fMRI findings were localized, AST could result in AD termination regardless of whether the site of AD initiation was in a region activated on fMRI.

**Figure 8:**
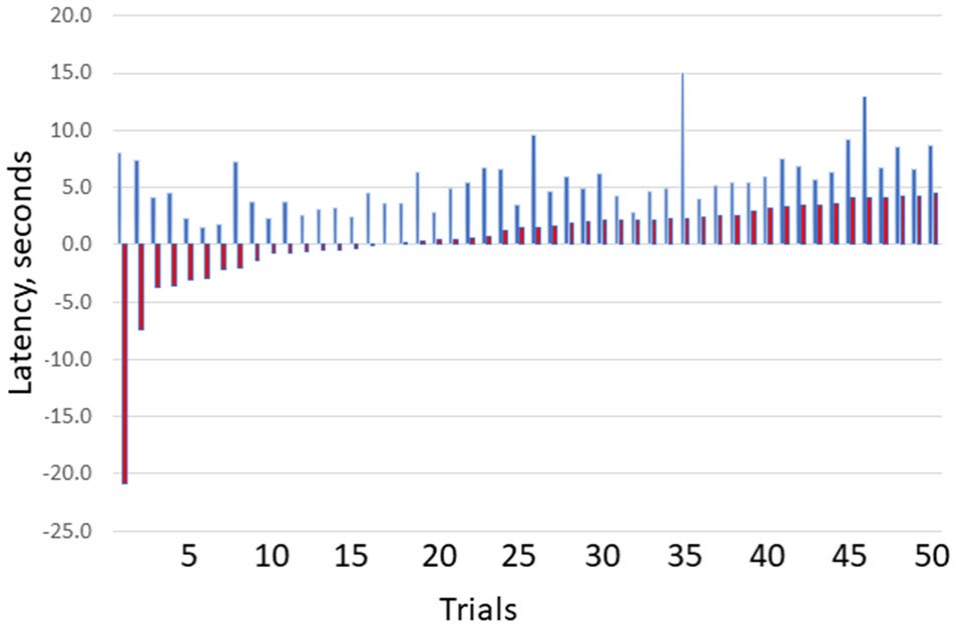
Question and answer latencies^6^. The figure shows the latency between (blue bars) question start and afterdischarge termination vs. (red bars) answer start and afterdischarge termination. As would be expected, ADs always ended after the question was asked. AD termination around the time that the patient answered the question would favor it being associated with factors related to the act of answering. However, there were no significant differences with respect to time: ADs ended throughout the trials, ending before an answer was initiated in 16/50 trials, as shown by the negative latencies for the first red bars. (16/50 trials = 32%. 95% CI 19.5%, 46.7%). X axis shows results for the 50 trials, with pairs sorted by increasing latency for (A). Y values are in seconds.

This suggests that functional localization per se is unlikely to account for all our findings. Instead, we suggest that ADs ended due to localized responses to diffuse changes. Bartolomei and colleagues have proposed that alteration in consciousness during seizures is due to synchronization among multiple and widespread cortical and subcortical regions, particularly the frontal, parietal, and cingulate regions^47,48^ Moreover, this group found that cognitive tasks, intense concentration, and focused attention were used successfully by patients to control their seizures^52^. They related these effects to the global workspace theory of consciousness which we discuss below. Our results are consistent with these conclusions but expand them: we found coherence changes throughout the entire cortex. In addition, we have shown that afterdischarge termination correlated with ongoing attention throughout the AST trial period, and not to the portion of the trial during which the patient answered the posed question^6^ (Fig. 8). We suggest that a common factor shared by cognitive performance, intense concentration, and focused attention is attention itself, present in the context of alertness and awareness. We agree with the previous authors that patients and their physicians might be able to use methods related to attention in helping to control at least some types of seizures. We also suggest that the effect of attention may be due to widespread decreases in synchronization or coherence, such as we report here. Although we used specific types of cognitive tasks, we think it unlikely that the particular task type is responsible for AD termination, and more likely that the task had a non-specific effect on attention and consciousness^42,53,54^.

With respect to treating intractable epilepsy, neither focal brain resection^55–57^ nor focal brain stimulation^58,59^ is always successful in completely controlling intractable epilepsy. Moreover, there is evidence that the effects and efficacy of both intraoperative and focal therapeutic brain stimulation may relate to changes distant to where stimulation occurred^1,60^. In addition to the importance of attention, our results suggest that in such cases it may be helpful to consider physiologic or pharmacologic treatments administered at times after the moment of electrical seizure onset and directed to locations, such as the middle distances reported here, that are located away from the presumed epileptogenic region. Perhaps mechanisms underlying methods such as thalamic and vagus nerve stimulation might in part involve changes in diffuse interactions that similarly go beyond the putative time and site of seizure onset. These considerations also might apply to other neurologic conditions.

### Attention and coherence

Our findings, related to coherence changes while the patients were attending to cognitive tasks therefore may have implications for the idea that mechanisms underlying conscious awareness localize to specific brain regions. There have been several models proposed to explain the cerebral underpinnings of attention and consciousness including integrated information theory, global neuronal workspace theory, and attention schema theory^47,61,62^. To some extent, each of these theories suggests specific brain sites that might importantly originate or control attention and consciousness. These sites include the prefrontal cortex, temporal-parietal region, cingulate cortex, and posterior cortex. However, many of these sites can be removed surgically, or altered due to strokes or tumors without altering attention and consciousness^63–68^.

Our studies of AD termination by AST show widespread changes (this paper as well as^4,5^) co-existing with more localized changes^3^. Specifically, ADs could be ended throughout the brain, regardless of whether the sites where stimulation produced ADs also participated in arithmetic or spelling^4^. Coherence decreases in the frequency ranges reported in our patients have previously been found in the setting of attention increases or increased cognitive load^69–76^. It is possible that AD termination was related to the patient answering the posed question, however, as described above, AD termination in response to AST was better correlated with attention to and consideration of the task request rather than the actual performance of the task^6^. More broadly, however, the quality of executive function, the capacity of working memory, and the level of patient engagement with the task and motivation for answering the posed question could be factors influencing whether ADs ended in response to the task setting^77^.

All of this, taken together, suggests that specific sites, regions, or networks may be important for, but disruption of any one of them may not invariably lead to, loss of attention or consciousness. We suggest an alternative: there may be several possible mechanisms underlying control of attention and consciousness. One mechanism may be a widespread change in coherence that cooperates with focal or multifocal mechanisms, with attention or consciousness occurring as an emergent property of the cortex (or brain) as a whole.

### Underlying pathophysiological processes

Small changes in extracellular field potentials can alter neuronal firing and neuronal synchrony^78^. We, therefore, speculate^4^ that the brain’s responses to the AST might constitute noise with respect to a previously stable and localized network underlying AD activity^3^, driving this network out of equilibrium^79^, with resultant termination of the ADs. Also, the task setting could alter activity in slow-inhibitory neurons^80–82^, which in turn could alter neuronal activity in the cortical pyramidal layers which are thought to generate alpha and beta range activity^70,73,83^.

### Limitations, potential confounders, and other considerations

An important limitation of this study is the small number of patients and trials, which may have limited statistical power for detecting differences. It would be helpful to confirm our findings in a larger group.

Another limitation relates to the amount of overlap between wavelets. However, a family of wavelets with less overlap between adjacent wavelets would be less sensitive to frequencies in the regions shared by two adjacent wavelets. For example, in Fig. 5, if two adjacent wavelets are separated from each other to a greater extent on the X axis, the value on the Y axis in the regions of overlap would be lower, resulting in lower sensitivity to activity in those regions. To create a family of wavelets with virtually no overlap and gaps between them, i.e. with steep box-like frequency response, we would need to use wavelets that would be several tens of seconds in duration and would be too long for this study.

In this study, we looked only at within-frequency coherence, but study of cross-frequency interactions might provide further insights into the phenomena we observed.

Our previous report showed that BPS, delivered to the same electrode contacts that caused the ADs can at times terminate these ADs^2^. Could transcranial stimulation provide an alternate, potentially less invasive, and potentially more effective, way of doing this? Modeling of transcranial stimulation showed that most of the current is shunted through the scalp^12,13^. Nonetheless, transcranial direct current stimulation can alter current flow through subdural strip electrodes. Therefore, despite the lower current density, this might be an alternative to BPS for terminating ADs. However, direct subdural stimulation of the area that produced the ADs is only effective in AD termination about half the time^2^, and we have found no significant difference in charge density between when BPS terminates ADs and when it does not^4^. One would have to test whether transcranial stimulation is more effective than this, or if it provides other advantages.

## Supplementary Information

Supplementary Table 1.

Supplementary Video 1.

Supplementary Video 2.

## Data Availability

The datasets generated and/or analyzed during the current study are not publicly available due to ethical and legal restrictions on sharing de-identified data that aligns with the consent of research participants. Current Johns Hopkins University compliance policies require data under waiver of consent to be shared under restricted access and not for commercial use. De-identified data will be deposited with an approved controlled access repository, for release with a Data Use Agreement to approved researchers: DABI repository https://dabi.loni.usc.edu. The dataset will be deposited as private under conditions of non-commercial research use, but are available from the corresponding author on reasonable request.

## Abbreviations

AD: Afterdischarge
ADs: Afterdischarges
AST: Arithmetic or spelling task
BPS: Brief pulse stimulation, brief pulses of electrical stimulation
CI: Confidence intervals
cm: Centimeters
dB: Decibel
ECoG: Electrocorticographic, electrocorticography
EEG: Electroencephalographic, electroencephalography
EDF: European data format
Eq: Equation
Hz: Hertz
mA: Milliamperes
µC: Microcoulombs
mV: Microvolts
Oct: Octave
RC: Resistance and capacitance, i.e. as in an electrical circuit
WxCoh: Wavelet cross-coherence
