# Supplementary material for: Pan-cortical electrophysiologic changes underlying attention
Source: Sci Rep. 2024 Feb 1;14:2680. doi: 10.1038/s41598-024-52717-w (PMC10834435; doi:10.1038/s41598-024-52717-w)
Supplement: Supplementary file 1 — Supplementary Table 1. [file 41598_2024_52717_MOESM1_ESM.pdf]

																			Arithmetic Results		Spelling Results		
**patient**	**gender**	**age onset**	**age surgery**	**seizure frequency**	**sz type**	**Wada language**	**Wada memory**	**handed**	**patient**	**AC Rx**	**surgery**	**lobe resected**	**imaging prior to this evaluation**	**FU Engel yrs**	**patient**	**electrodes implanted**	**electrodes monitored**	**combinations analyzed**	**M1**	**M0**	**S1**	**S0**	**SUM**
**S1**	f	21y	41y	2/m	FA FI	L	B	R	**S1**	OXC	L F res	F	L mes T ↑ T2, L mes T↓ size	IA / 12	**S1**	91	86	3655	18 / 7	14 / 3	2 / 2	2 / 2	36 / 14
**S2**	m	16y	30y	2/d	FA FB			R	**S2**	LTG	R P les	P	R postcentral sulcus cortical dysplasia	IA / 7	**S2**	81	73	2628	5 /4	5 / 3			10 / 7
**S3**	f	3m	20y	1-sev/m	FA			R	**S3**	LTG ZNS	R F les	R	R F focal dysgenesis	IA / 7	**S3**	88	82*	3321	5 / 5	3 / 3			8 / 8
**S4**	m	10y	16y	3-4/d	FA FI			R	**S4**	VPA	L T les	L	L T calcified mass	?	**S4**	61	56	1540	1 / 0	3 / 0	2 / 0	4 / 0	10 / 0
**S5**	m	8 h	12 y	sev/m	FA FI FB			L>R	**S5**	VPA LOR	rt post F les	F	R precentral encephalomalacia	IIA / 9	**S5**	81	75*	2775	2 / 0				2 / 0
**S6**	m	46y	50y	1/w	FI FB	L	B	L>R	**S6**	CBZ	L B res, leave H	T	L T ↑ FLAIR T2	IA / 9	**S6**	97	91	4095	1 / 0				1 / 0
**S7**	f	10y	27y	up to 60/d	FA FB			R	**S7**	LTG	Ant RF re-res	F	ictal SPECT – RF,RI uptake. PET 20y min ↓ RT, 21y- ↑ RF, RI	IIIA / 7	**S7**	101	99	4851	3 / 2	2 / 2			5 / 4
**S8**	f	7y	53y	2-3/w	FA FI FB	L	L	R	**S8**	LEV CBZ	R T re-res	T	R H ↑ T2, ↓ size	IIIA / 10	**S8**	62	59	1711	1 / 0	3 / 0		1 / 0	5 / 0
**S9**	f	12y	32y	3-5/w	FI FB	L	L	R	**S9**	PHN	extend RT res	T	R periH cyst	IA / 6	**S9**	83	79	3081	1 / 0				1 / 0
**S11**	m	32y	41y	daily	FI	L	B, L>R	R	**S11**	VPA CBZ	L T Lob sparing H	T	L H ↑ FLAIR. LT PET ↓ FDG, multiple aneurysms	IC / 9	**S11**	81	77	2926	1 / 0				1 / 0
**S12**	f	47y	50y	up to 10/d	FA FI	L	R	R	**S12**	OXC PGB	L T Lob sparing base & H	T	L FT operculum/insula oligodendroglioma	IB / 8	**S12**	108	105*	5460	2 / 1	1 / 1			3 / 2
**S14**	m	8y	14y	qod	FI			R	**S14**	OXC RUF LCM VNS	deferred. Subsequent les	O	L mesial occipital closed lip schizencephaly; R thalamus ↑ T2FLAIR	IA / 5	**S14**	96	88	3828		3 / 0	6 / 0	10 / 2	19 / 2
summary	6m 6f	8h - 46 y	12-53 y													1030	708	39871	40 / 19	34 / 12	10 / 2	17 / 4	101 / 37

ABBREVIATIONS

patient S1-15 indicates patient # 1-15

gender f=female, m=male

age onset, age surgery, seizure frequency h=hours, d=day, w=week, m= month(s), y=years, sev=several, qod=every other day

Sz type (seizure type) FA=focal aware FI=focal impaired awareness FB=focal to bilateral tonic-clonic, using the terminology suggested by the International League Against Epilepsy Commission for Classification and Terminology*

*Fisher RS, Cross JH, French JA, Higurashi N, Hirsch E, Jansen FE, et al. Operational classification of seizure types by the International League Against Epilepsy: Position Paper of the ILAE Commission for Classification and Terminology. Epilepsia 2017; 58(4): 522-30.

Wada (if performed), handed handed=left/right handed L=left dominant, R=right dominant, B=bilateral representation, L>R = left predominance

AC RX anticonvulant treatment at time of surgery

CBZ=carbamazepine, LCM=lacosamide, LEV=levetiracetam, LOR=lorazepam, LTG=lamotrigine, OXC=oxcarbazepine, PGB=pregabalin, PHN=phenytoin, VNS=vagal nerve stimulator VPA=divalproex, ZNS=zonisamide

Surgery lobe resected Imaging prior to this evaluation

Surgery=surgery performed consequent to this evaluation.

A = anterior P= posterior L= left R= right B= temporal base F = frontal I=insula mes=mesial P=parietal T=temporal H= hippocampus MCA= middle cerebral artery distribution

Les= lesionectomy Lob=lobectomy Res=resection Re-res = re-resection prev=previous.

↑ ↓ = increased or decreased signal on imaging study. T2=T2 weighted magnetic resonance imaging study, FDG=fluorodeoxyglucose PET study, FLAIR=Fluid-attenuated inversion recovery magnetic resonance imaging study

MRI=magnetic resonance imaging PET=positron emission tomography SPECT=single-photon emission computerized tomography if not specifically labeled, imaging findings are those from MRI

FU Engel* yrs Outcome on follow-up using Engel criteria / years since surgery at follow-up. ? = lost to follow up

*Engel Jr J. Outcome with Respect to Epileptic Seizures. In: Engel Jr J, editor. Surgical Treatments of the Epilepsies. New York: Raven Press; 1987. p. 553-71.

electrodes implanted = number of implanted electrodes in this patient; **electrodes monitored** = electrodes actually monitored. Others were ground electrodes or malfunctioned

combinations analyzed - total number of pair combinations analyzed. See section 2.6 of report for further details.

M1, M0, S1, S0 = Math (M) or Spelling (S) problem followed (M1, S1) or not followed (M0, S0) by termination of ADs.

X / Y = Total trials / trials during which there was no brief pulse stimulation (BPS) prior to the Math (M) or Spelling (S) problem

Supplementary Table - information regarding the 12 patients

Modified from Lesser et al. Clinical Neurophysiology 130:2169-2181, 2019. with permission from and published by Elsevier B.V. and ©International Federation of Clinical Neurophysiology 2019.

Patients S10, S13, and S15: afterdischarges did not stop in response to the cognitive tasks and so their information is not shown here. It is in the 2019 publication.

Please note that there were errors regarding electrodes implanted an monitored in the 2019 publication of this table, which are corrected herein, and also at https://doi.org/10.1016/j.clinph.2021.12.009
